# Quantitation of Vacuolar Sugar Transporter Abundance Changes Using QconCAT Synthtetic Peptides

**DOI:** 10.3389/fpls.2016.00411

**Published:** 2016-04-12

**Authors:** Heidi Pertl-Obermeyer, Oliver Trentmann, Kerstin Duscha, H. Ekkehard Neuhaus, Waltraud X. Schulze

**Affiliations:** ^1^Department of Plant Systems Biology, University of HohenheimStuttgart, Germany; ^2^Plant Physiology, University of KaiserslauternKaiserslautern, Germany

**Keywords:** vacuole, sugar transport in plants, salt stress, drought stress, QconCATs

## Abstract

Measurements of protein abundance changes are important for biological conclusions on protein-related processes such as activity or complex formation. Proteomic analyses in general are almost routine tasks in many laboratories, but a precise and quantitative description of (absolute) protein abundance changes require careful experimental design and precise data quality. Today, a vast choice of metabolic labeling and label-free quantitation protocols are available, but the trade-off between quantitative precision and proteome coverage of quantified proteins including missing value problems remain. Here, we provide an example of a targeted proteomic approach using artificial standard proteins consisting of concatenated peptides of interest (QconCAT) to specifically quantify abiotic stress-induced abundance changes in low abundant vacuolar transporters. An advantage of this approach is the reliable quantitation of alimited set of low-abundant target proteins throughout different conditions. We show that vacuolar ATPase AVP1 and sugar transporters of the ERDL (early responsive to dehydration-like) family and TMT2 (tonoplast monosaccharide transporter 2) showed increased abundance upon salt stress.

## Introduction

Measurements of protein abundance changes allow conclusion about post-transcriptional regulatory processes, for example through correlation with protein activity or complex stoichiometry. Quantitative proteomic analyses are routine tasks in many experiments, and a large selection of methods using metabolic labeling or label-free approaches is available reviewed in Schulze and Usadel (2010) and Arsova et al. (2012a). However, although, for yeast full proteome coverage has been claimed (Walther et al., 2010) and up to 10000 proteins were identified from human tissue in single LC–MS/MS runs (Mann et al., 2013), even with modern, fast, and highly accurate mass spectrometers the full proteome measurements are not yet routinely feasible for most labs. Particularly if tissues with highly skewed protein abundance distributions are analyzed, such as plant leaf tissues (Arsova et al., 2012b), uniform protein coverage becomes more challenging. Furthermore, membrane proteomics is still rather pretentious and thus large scale proteomic data sets still are often not complete and particularly lacking information for low abundant membrane proteins such as ion channels and certain transporters.

A common workflow to address this problem is to firstly perform profiling experiments, then, secondly to define a list of relevant target proteins/peptides which will then be studied using dedicated targeted protein analysis methods (Schulze, 2010). Such workflows were successfully used to study protein phosphorylation responses under osmotic stress (Stecker et al., 2014) or to study specific enzyme activities and their regulatory phosphorylation sites (Glinski and Weckwerth, 2005). In yeast, the central carbon and amino acid metabolism was in detail studied by a targeted approach (Costenoble et al., 2011). Most of these targeted approaches require the use of triple quadrupole or quadrupole-Orbitrap mass spectrometers and synthetic versions of the target peptide sequences for tuning and as internal quantitation standards (Gallien et al., 2012; Kelstrup et al., 2012). Setup of such targeted experiments is quite challenging as often redundancy occurs in precursor and product ions resulting in lower quantitation specificity of the selected targets (Sherman et al., 2009). As an alternative strategy, and to avoid expensive synthesis of synthetic peptides, the concatenated peptides (QconCAT) were developed allowing the expression of concatenated peptides as an artificial protein in bacteria (Pratt et al., 2006). These artificial proteins can easily be stable-isotope labeled in bacteria and then be digested to obtain tryptic standard peptides for mass spectrometric analysis and quantitation. Heavy labeled peptides are added to the sample of interest in known quantities and ion intensities are measured for the light peptides, originating from the biological sample, and for the heavy peptides, representing the standards. Finally, their ratio of the respective heavy and light signal intensities can be used to calculate the absolute quantity. Such labeled peptides were used in various studies quantifying developmental changes in muscle cells (Rivers et al., 2007) or in studying the glycolytic pathway in yeast (Carroll et al., 2011). In plants, spiked in digested peptides originating from a QconCAT synthetic protein were used to study the stoichiometry in plastidial Clp protease complexes (Olinares et al., 2011), but have not yet been widely used.

The vacuole is an important plant specific organelle with functions in storage of solutes as nutrient reservoirs, but also with important roles in adaptation to stresses, such as cold stress (Wormit et al., 2006; Schulze et al., 2012), salt stress or drought (Rizhsky et al., 2004; Hedrich et al., 2015). Various transporters for solutes into and out of the vacuole were identified and individually characterized in their physiological context (Martinoia et al., 2012). Sugars play important roles as nutrients as well as signal molecules or compatible solutes during stress. Particularly, in the recent years vacuolar sugar transporters were characterized in detail. They are mainly membrane proteins belonging to the major facilitator superfamily (MFS) whose subfamilies of sucrose transporters (SUTs) and monosaccharide transporters (MSTs) are well-studied also under drought stress (Medici et al., 2014). In that context, the family of tonoplast monosaccharide transporters (TMTs) was characterized as glucose transporters involved in the re-distribution of cellular glucose to the vacuole under cold stress conditions (Wormit et al., 2006). More recently, TMT1 and TMT2 were also found to transport sucrose (Schulz et al., 2011). Another family of vacuolar sugar transporters (VGT/ERD) contains importers and exporters of vacuolar sugars (Büttner, 2007). Besides glucose, fructose and sucrose, also sugar alcohols like sorbitol, mannitol, and myo-inositol are transported into vacuoles, suggesting a fine-tuning of the transport activities across the tonoplast dependent on external conditions and nutrient supply.

There is evidence that in *Arabidopsis* various abiotic stresses, especially cold stress, lead to accumulation of sugars, particularly glucose and fructose in the vacuole (Wormit et al., 2006; Schulze et al., 2012). An increased accumulation of sugars upon drought and heat stress has also been observed (Rizhsky et al., 2004) suggesting a role of vacuolar sugar transporters also under these conditions. Expression of the putative sugar transporter ERD6 (early responsive to dehydration) is induced not only by dehydration but also by cold treatment (Kiyosue et al., 1998), and expression of an ERD6-like transporter (ESL1) is enhanced by drought, salt, and ABA treatment (Yamada et al., 2010). Osmotic stress and salt stress also affects vacuolar transporters, particularly the sucrose transporter SUC4 (Gong et al., 2015) and the v-ATPases (Kirsch et al., 1996).

All of these energy-driven fluxes of metabolites across the tonoplast are channeled by a range of different transporters. Therefore, in addition to sugar transporter, in a typical tonoplast also energizing transporters are found, such as the V-PPase, the V-ATPase, ABC transporters and Ca^2+^ pumps. During salt stress the activity of the vacuolar H^+^ pumps is increased and accompanied also by induced gene expression (Hasegawa et al., 2000; Maeshima, 2001). In that context, it has been demonstrated that overexpression of the H^+^-PPase AVP1 increases salinity tolerance in *Arabidopsis* (Gaxiola et al., 2001). In addition there are several transporters for water and organic solutes, anion channels and cation transporters like potassium channels and zinc or copper transporter (Martinoia et al., 2007). Recently it became obvious that the vacuole as such, or the regulation of tonoplast transporter, plays an important role during adaption to abiotic environmental stress.

Here, we developed and applied a targeted quantitative proteomics workflow using QconCAT synthetic standard peptides to study particularly the abundance changes of sugar transporters in the tonoplast-enriched fractions from *Arabidopsis* leaves grown under abiotic stress conditions such as salinity or drought.

## Results

### Determination of the Linear Range of Quantitation

**FIGURE 1:**
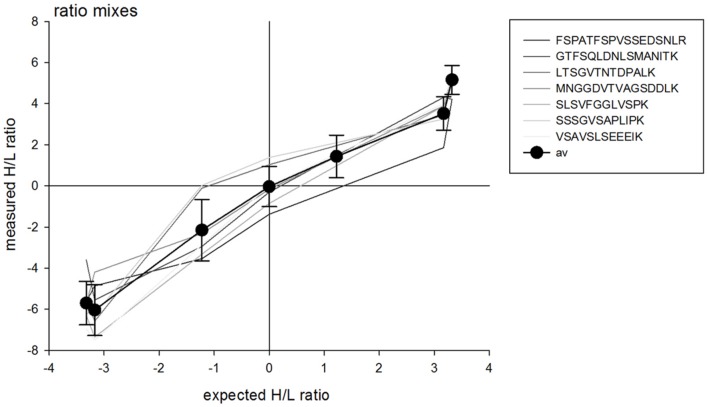
**Measured heavy (H) to light (L) ratio of different mixing ratios of labeled (H) and unlabeled (L) QconCAT.** A total of 5 μg was injected for each mixture. Differently shaded lines indicate the measured ratios for individual peptides of the QconCAT, black circles indicate the averages across all peptides with standard deviation.

Unlabeled (L, light) and labeled (H, heavy) QconCATs were mixed in different ratios (L:H ratios of 10:0, 9:1, 7:3, 5:5, 3:7, 1:9, and 0:10) to result in a total of 10 μg of protein before tryptic digest. After tryptic digestion and desalting, 5 μg of each mixture was analyzed by LC–MS/MS. The measured H/L ratio of each mixture of labeled and unlabeled peptides showed a linear relationship with the expected H/L ratio provided by the peptide mixture (**Figure 1**). Only in very low H/L mixing ratios, the measured ratios were lower than expected suggesting that under these conditions, the signal to noise ratio of the full scan spectra did not fully resolve the very low presence of unlabeled peptide. Different peptides showed slightly different curves reflecting variations in ionization properties.

### Detection of Spiked-in Peptides in Complex Sample Background

**FIGURE 2:**
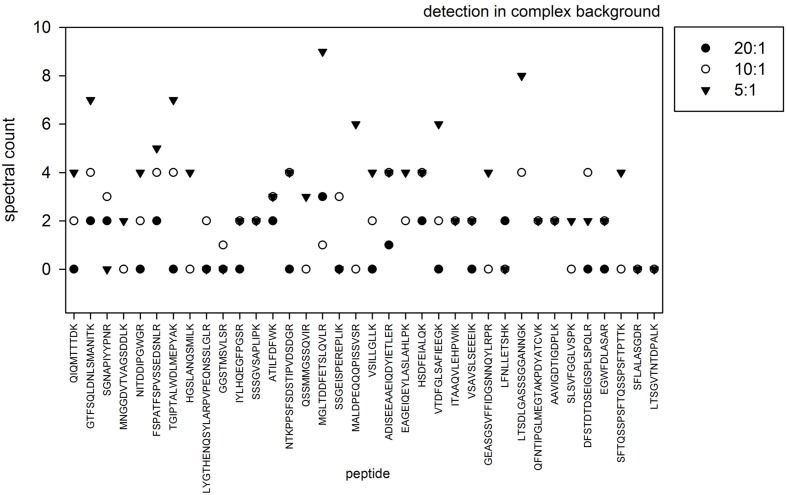
**Spectral count of different peptides resulting from QconCAT digestion measured in the background of 5 μg (triangle), 10 μg (open circle), and 20 μg (filled circle) of microsomal protein**.

To determine the limit of detection within a complex protein background, labeled QconCAT protein was spiked into different amounts of microsomal protein preparations resulting in w/w protein:QconCAT ratios of 5:1, 10:1, and 20:1. After joint tryptic digestion and desalting of these spiked protein mixtures, 5 μg of each mixture was analyzed. As expected, under conditions of the QconCAT protein being mixed with lower amounts of the microsomal protein, higher spectral counts were achieved for each of the QconCAT peptides resulting from the tryptic digestion (**Figure 2**). Based on these results, in further experiments a mixing ratio or protein:QconCAT of 5:1 was used to ensure efficient coverage of the target peptides in the mass spectrometric analysis.

### Reproducibility: Variations in Intensities, Retention Time, and Spectral Counts

**FIGURE 3:**
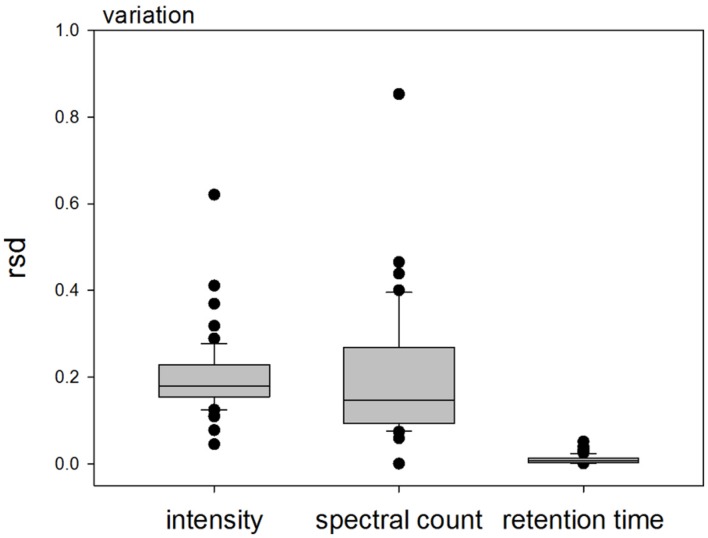
**Relative standard deviation of detected ion intensities, spectral counts and retention times within each QconCAT peptide after multiple injections of 5 μg digested QconCAT**.

Next, we analyzed the reproducibility of QconCAT detection. Thus, a total of 25 μg of QconCAT protein was in-solution digested, desalted and injected five times in 5 μg injections for mass spectrometric analysis. The five injections were done either from different wells on the autosampler plate or repeatedly sampled from the same well. To determine the reproducibility between runs, detected peptides were analyzed according to variation in peak intensities, retention times and the number of recorded spectra. Across all peptides and the five injections, retention time variations from run to run was extremely low. In contrast the ion intensity variation averaged around 0.2 rsd, and the spectral counts showed largest variation (**Figure 3**). These results confirm that our chromatography and subsequent retention time alignment of raw data is very reproducible and that ion intensity (or ion intensity ratio) quantitation is more robust and less prone to variation than spectral counting.

### Large Alterations in Abundance of Vacuolar Sugar Transporters under Salt and Drought Stress

**FIGURE 4:**
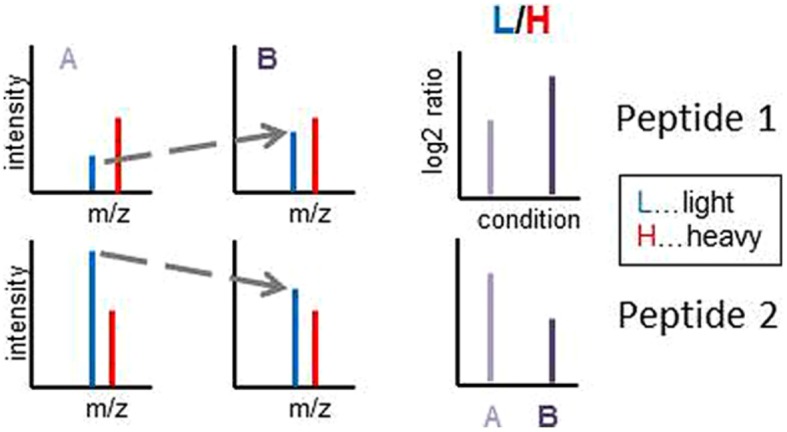
**Illustration of the quantitation workflow using the L/H ratios of endogenous plant-derived light peptides (L) and the QconCAT-derived isotope labeled standard peptides (H).** In the spiking experiments, it will be important that the amount of the spiked-in standard (H, heavy) needs to be added in the same amount in all experiments/conditions to be compared. This is indicated by equal peak intensities in the illustrations.

The QconCAT protein was spiked into vacuolar preparations of plants grown under salt stress or drought stress and respective control conditions. We were particularly interested in following the changes in protein abundance of several vacuolar sugar transporters which play an important role in adjustments of cellular solute concentrations under these stress conditions. The abundance changes in sugar transporter abundance were based on changes of proteotypic signature peptide ratios in stressed plants compared to their control conditions (**Supplementary Table S1**). The peptides were chosen with high probability for good ionization properties (Brownridge et al., 2011) or based on experimentally identified peptides in previous work (Schulze et al., 2012) or from public databases (Joshi et al., 2011). We then used the ratio of light (L, unlabeled) plant-originating peptide version to the heavy (H, labeled) peptide originating from the spiked-in QconCAT for quantitation (**Figure 4**). Using the L/H ratio, with constant amount of the heavy standard peptide (H), the ratio directly is proportional to the abundance ratio of plant-derived peptide (L) compared to its standard. Comparing two different conditions (stress treatment and control), the change in L/H ratio depicts the change in protein abundance induced by the stress condition (**Figure 4**) and the ion intensities of the spiked-in standard can be used for normalization between different samples. Thus, a change of the L/H ratio to higher (more positive) values is proportional to an increase in abundance of the peptide (and protein) while a ratio change to lower (more negative) values is proportional to a decrease of peptide and protein abundance (**Figure 4**).

The amount of the spiked in standard protein was 1 μg, but after tryptic digestion within the mixture with the vacuolar protein, each standard peptide was produced to different absolute amounts according to the molecular weight of each peptide, ranging from 10 to 34 ng in the final digested 5:1 mixture of protein:QconCAT (**Supplementary Table S1**). However, each peptide was produced to the same molar amount: the 1 μg spiked-in QconCAT corresponded to 13 fmol of protein. With knowledge of the molar amount of standard added to the sample, the molar amount of each peptide can be calculated as follows: L/H * (m_QconCAT_/MW_QconCAT_). Such calculations revealed AVP1 as the most abundant protein with 45 ± 31 mmol/g_protein_ and VMA21a and VMA22 as the least abundant proteins with 0.3 ± 0.12 mmol/g_protein_. Among the sugar transporters, ERDL4 and TMT2 were most abundant averaging 24 ± 3.8 mmol/g_protein_ and 5.8 ± 2.5 mmol/g_protein_, respectively.

**FIGURE 5:**
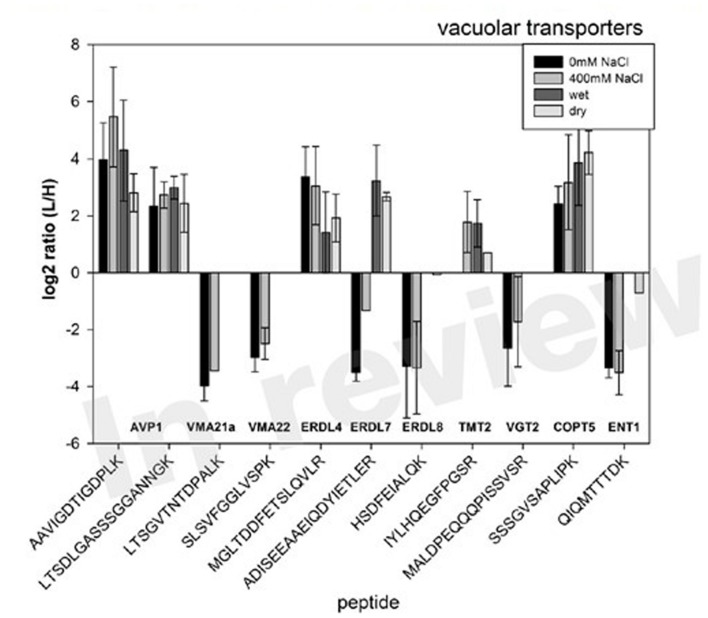
**Selected examples of L/H ratio of peptides matching various vacuolar transporters, particularly the members of the ERD and TMT family under salt stress and drought condition relative to their control conditions.** Bars are averages of three biological replicates with standard deviation.

The L/H ratios in control conditions, salt stress (400 mM NaCl) as well as control and drought conditions could be derived for several vacuolar transporters (**Figure 5**). Thereby, in some cases, such as for high abundant proteins AVP1 (AT1G15690), ERDL4 (AT1G19450) and COPT5 (AT5G20650), the endogenous peptides (L) was in all cases more abundant than the spiked in standards (H) leading to large L/H ratios across conditions. In other cases, such as for low abundant VMA21a (AT2G31710), VMA22 (AT1G20770), ERDL7 (AT2G48020), and ERDL8 (AT3G05150), the endogenous peptides (L) were less abundant than the spiked-in standard (H) leading to low L/H ratios across conditions.

Under salt stress, we observed an increase of protein abundance for most of the monitored proteins. Particularly, for AVP1 (AT1G15690) abundance significantly increased almost threefold when plants were exposed to salt stress of 400 mM NaCl (**Table 1**). Among the sugar transporters, significant and large increases in abundance were observed also for ERDL7 (AT2G48020) and TMT2 (AT4G35300). ERDL4 (AT1G19450) and ENT1 (AT1G70330) were the only sugar transporter for which no significant change in abundance was measured under salt stress. Under drought stress, for most proteins a mild decrease in protein abundances was observed (**Table 1**). These mild changes were significant only for AVP1 (AT1G15690), ERDL7 (AT2G48020), and ENT1 (AT1G70330) which decreased from vacuolar membranes. A significant drought-induced increased abundance was observed for ERDL4 (AT1G19450) and COPT5 (AT5G20650).

**Table 1:** Summary of changes in protein amounts under salt and drought stress comparing QconCAT quantitation with label-free quantitation.

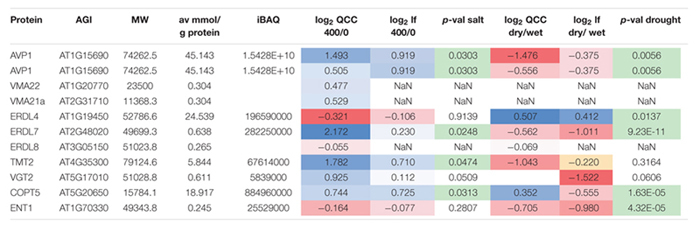

*Red and blue indicate down and up regulated protein expression, respectively. Color intensity is proportional to the intensity of change. Significant changes (*p*-value < 0.05, *t*-test) are shaded in green.*

The measured abundance changes using the QconCAT-derived standard peptides were confirmed by an independent label-free quantitation using the same raw files. The very low abundant proteins VMA21a (AT2G31710), VMA22 (AT1 G20770), and ERDL8 (AT3G05150) could not be quantified in the label-free analysis. Overall, in the label-free analysis the abundance changes resulted in smaller amplitudes for some proteins, but with similar trends as in the ratio quantitation (**Table 1**). Only for COPT5 (AT5G20650) under drought stress, an increased abundance was measured based on the QconCAT quantitation while the label-free quantitation resulted in a decreased abundance.

## Discussion

In this work we explored the use of QconCAT standard proteins to enhance the quantitation of specific target proteins, such as vacuolar transporters. Thereby, we firstly provide some technical properties on quantitation range, detection limit and reproducibility. Technically, we co-digested vacuolar protein together with the spiked-in standard protein. We specifically decided against alternative to separately digest the standard protein and then add defined amounts of the resulting standard peptides to digested vacuolar protein preparations in order to more precisely spot sample-to-sample variations. 24 peptides (out of 49 peptides within QconCAT) were reproducibly detected in replicates of control and stress treatment samples allowing solid quantitation in both conditions.

In the past years, several methods for absolute protein quantitation strategies were developed, such as emPAI (Ishihama et al., 2005), APEX (Braisted et al., 2008), the TOP3-method (Silva et al., 2006), or iBAQ (Schwannhäusser et al., 2011) which all have in common that they allow protein abundance ranking without the use of spiked-in internal standards. These methods are based on several peptides identified for each protein or on the most optimal three ions as in the TOP3-method. The emPAI values were successfully used to calculate cellular concentrations of metabolic enzymes (Arrivault et al., 2014), and a collection of spiked-in synthetic standard peptides was used to quantify Calvin cycle enzymes in Chlamydomonas (Wienkoop et al., 2010).

The absolute quantitation of protein amounts using spiked-in standard peptides is most likely to be less accurate due to lower statistical power of only few peptides, and these peptides may not necessarily be among the most optimal peptides which otherwise are for iBAQ or TOP3 quantitation. It has been shown that quantitation using standard peptides particularly depends on good design of the peptides (Brownridge et al., 2011), and experimental evidence for the chosen peptides from previous analyses is beneficial. Thus, not all peptides are equally suitable to quantify the absolute protein amounts due to low ionization properties or modification sites. The peptides used here were designed to allow clear identification of the target proteins (proteotypic peptides) and in some cases, such as for AVP1 or ENT1 one of the three peptides designed could not be avoided to be cysteine containing due to structural constraints (i.e., avoiding transmembrane spans). In general, the absolute protein amounts calculated here corresponded well (*r* = 0.89, *r*^2^ = 0.81) with the iBAQ values calculated from a label-free analysis.

The observation that TMT2 protein abundance decrease during drought is surprising, given the high monosaccharide import specificity of latter carrier (Wormit et al., 2006) and given drought conditions provoke accumulation of sugars in leaves of higher plants (Krasensky and Jonak, 2012). However, it might be that during drought monosaccharides have to accumulate in other compartments of the cell. This assumption is based on the observation that sugars act as efficient ROS scavengers (Lineberger, 1980) and it is widely known that under abiotic stress ROS generation takes especially place in chloroplasts, peroxisomes and mitochondria (Cruz de Carvalho, 2008). Furthermore, general protein degradation processes during drought stress could also reduce the abundance of these transporters in the tonoplast.

From none of the ERDL proteins mentioned (**Table 1** and see Results) the transport characteristics have been determined (Hedrich et al., 2015). Thus, it appears unjustified to interpret the changes in protein abundancies of ERDL4 and ERDL7 in response to salt or drought on a mechanistic level. This interpretation becomes even more complex given the known post-translational modification of TMT-type proteins with consequences on transport activity (Wingenter et al., 2011). In other words, the observed changes of ERDL protein abundancies have in the near future to be correlated with exact transport properties (e.g., substrate specificities and modes of transport) and with a detailed search for putative post-translational modifications.

In conclusion, the QconCAT standard peptides are very useful in experiments where a defined set of target proteins is to be analyzed, as for example in this study the focus was specifically on vacuolar (sugar) transporters. We demonstrated that the reproducibility of QconCAT detection is very high and that ion intensity ratio quantitation is more sensitive and more and robust than spectral counting.

The use of these spiked-in standards clearly does exceed the range of quantitation to include more low-abundant proteins compared to a simple label-free experiment. We showed that salinity stress results in large changes in vacuolar transporter abundances, particularly for vacuolar ATPases AVP1, members of the ERDL-transporter family as well as for the monosaccharide transporter TMT2. Since salinity is known to increase the sugar content in vacuoles (Rizhsky et al., 2004), we suggest that this increase in vacuolar sugar content is achieved by an increase in transporter presence in the tonoplast.

## Materials and Methods

### Bacterial Strains and Used Vector

Competent *Escherichia coli* BL21 (DE3) with the genotype B F^-^*dcmompThsdS*(r_B_^-^m_B_^-^) *gal*λ(DE3) were purchased from Agilent Technologies. The selected QconCAT peptides (see **Supplementary Table S1**) were concatenated and flanked by a leader N-terminal sequence (MAGK) and a C-terminal sequence (LAAALEHHHHHH) for purification (Polyquant, Bad Abbach, Germany). The full QconCAT1 protein sequence is:

MAGKCLTAVFMLEDEKATILFDFWKHKPDASFVAEAKSLS VFGGLVSPKFSPATFSPVSSEDSNLRQSSMMGSSQVIRHGSLA NQSMILKQTTSMDKGVVALDLGRLYGTHENQSYLARPVPEQ NSSLGLREGWFDLASARDFSTDTDSEIGSPLSPQLRGTFSQLD NLSMANITKHKPDAAFLAEAKLFNLLETSHKDNDDYATDDG AGDDDDSDNDLRLLLMISSIGMTISLVIVAVAFYLKSSGEISPER EPLIKSGNAPIYYPNREATFGELFRITAAQVLEHPWIKSFTQSS PSFTQSSPSFTPTTKTGIPTALWDLMEPYAKVSAVSLSEEEIKM NGGDVTVAGSDDLKMGLTDDFETSLQVLRGATSSDDHALK VTDFGLSAFIEEGKNITDDIPGWGRLTSGVTNTDPALKLAMG PLCDLIGPRADISEEAAEIQDYIETLERSFLALASGDRGGSTMS VLSRQIQMTTTDKMALDPEQQQPISSVSRSSSGVSAPLIPKNT KPPSFSDSTIPVDSDGRQFNTIPGLMEGTAKPDYATCVKGEA SGSVFFIDGSNNQYLRPRLTSDLGASSSGGANNGKIYLHQEGF PGSRRPFIHTGSWYREIGTGNIYACKVSILLGLLKEAGEIQEYL ASLAHLPKAAVIGDTIGDPLKDPLVNLFGSLHEKHSDFEIALQ KLAAALEHHHHHH.

The plasmids encoding the proteins for QconCAT1were cloned into the *Nde*I/*Bam*HI restriction sites of pET21a (Novagen, Nottingham, UK). A list of tryptic peptides and corresponding proteins they were derived from is shown in **Supplementary Table S1**.

### Expression, Stable-Isotope Labeling, and Purification of QconCAT1

Subsequent transformation, protein expression, and purification were performed as described in Pratt et al. (2006). In brief, the plasmid carrying the artificial gene for QconCAT1 was introduced into chemically competent *E. coli* using a standard transformation protocol, and frozen stocks were stored at -80°C. The QconCAT protein was expressed in *E. coli* cultures with a full complement of unlabeled amino acids (M9 minimal medium Sambrook et al., 1989) or in the presence of [^13^C_6_]arginine (10 mg 100 ml^-1^) and [^13^C_6_]lysine (10 mg 100 ml^-1^). Expression was induced with IPTG (isopropyl ß-D-thiogalactopyranoside), and after 5 h the cells were harvested by centrifugation at 4,000 rpm for 15 min at 4°C. Inclusion bodies containing the QconCAT were recovered by breaking the cells using BugBuster Protein Extraction Reagent (Novagen, Nottingham, UK) and after several washing steps centrifuged at 16,000 × *g* for 15 min at 4°C. For purification of the labeled and unlabeled QconCATs, the pellets of inclusion bodies were resuspended in IMAC binding buffer (20 mM phosphate buffer, pH 7.4, 500 mM NaCl, 20 mM imidazole, 6 M guanidine hydrochloride) and loaded onto gravity flow Ni^2+^-NTA sepharose columns (1 ml, IBA GmbH, Goettingen, Germany). After several washing steps the bound QconCAT was eluted with elution buffer (20 mM phosphate buffer, pH 7.4, 500 mM NaCl, 500 mM imidazole, 6 M guanidine hydrochloride) in 5 ml × 1 ml fractions. Purified QconCATwas desalted by dialysis (Slide-A-Lyzer^TM^ Dialysis Cassettes, 3,500 MWCO, 3–12 ml, Pierce) against 100 volumes of 50 mM ammonium bicarbonate, 1 mM DTT for 3 h × 2 h at 4°C. Finally, the protein content was determined using BSA as a standard (Lowry DC, Bio-Rad, Vienna, Austria). To ensure stability, purified QconCAT was aliquoted into 10, 25, 50, and 100 μg samples and dried down in a speed vac to completely dryness and stored at -80°C. Before use, QconCAT protein was resuspended in 6 M urea, 2 M thiourea, pH 8 in Tris-HCl to result in a final concentration of 1 μg μL^-1^.

### In-solution Digest for Mass Spectrometry

For in-solution digest the purified QconCAT protein (5 or 10 μg) was denatured using UTU buffer (6 M urea, 2 M thiourea, pH 8.0). After reduction in 6.5 M DTT and alkylation of cysteine residues by 27 mM iodoacetamide, proteins were digested for 3 h by LysC (Wako, Japan) at room temperature. The solution was then diluted fourfold with 10 mM Tris-HCl, pH 8.0 followed by overnight digestion with trypsin (sequencing grade, Promega) at 37°C at 350 rpm. Finally, digested peptides were desalted over C18 STAGE- tips and dried down in a speed vacuum concentrator and stored at -80°C. For mass spectrometric analysis samples were resuspended in resuspension buffer (0.2% v/v TFA, 5% v/v acetonitrile).

Tryptic peptide mixtures were analyzed by LC–MS/MS using nanoflow HPLC (Proxeon Biosystems, Denmark) and a hybrid quadrupole-orbitrap mass spectrometer (Q Exactive Plus, Thermo Scientific) as a mass analyser. Peptides were eluted from a 75 μm × 15 cm C18 analytical column (PepMap^®^ RSLC C18, Thermo Scientific) on a gradient using 0.5% acetic acid as aqueous phase and 0.5% acetic acid in 80% acetonitrile as organic phase. The flow rate was set to 250 nL per minute. Peptides were eluted on a linear gradient running from 4 to 64% acetonitrile in 135 min. Spectra were using information-dependent acquisition of fragmentation spectra of multiple charged peptides within the m/z range of 300–1600. Up to 12 data-dependent MS/MS spectra were acquired for each full-scan spectrum acquired at 70,000 full-width half-maximum resolution. Fragment spectra were acquired at a resolution of 35,000. Masses of peptides of QconCAT1 were used as inclusion list to induce preferred fragmentation of the target peptides of interest.

Protein identification and ion intensity quantitation was carried out by MaxQuant version 1.4.1.2 (Cox and Mann, 2008). Spectra were matched against the *Arabidopsis* proteome (TAIR10, 35386 entries) using Andromeda (Cox et al., 2011) and the following search parameters: multiplicity was set to 2, Arg6 and Lys6 were selected as heavy labels; carbamidomethylation was selected as a fixed modification for cysteines, methionine oxidation was selected as variable modification, two missed cleavages were allowed. Precursor mass tolerance was set to 20 ppm and MS/MS tolerance to 0.5 Da. Peptides were accepted with a length of more than seven amino acids under a protein match false discoveryrate threshold of 0.01, peptide spectral match false discovery rate threshold of 0.01. Retention time alignment was done in a window of 2 min. Quantitation of spiked-in heavy QconCATs and internal unlabeled peptides from the plant extract was performed using the SILAC heavy/light quantitation within MaxQuant.

### Preparation of Microsomal Protein

Microsomal fractions (MFs) of seedling cultures were prepared by differential centrifugation (Pertl et al., 2001). Frozen *Arabidopsis* seedlings (approximately 20 g of fresh weight) from Col-0 were smashed into small pieces and resuspended in ice-cold homogenisation buffer (330 mM sucrose, 100 mM KCl, 1 mM EDTA, 50 mM Tris adjusted with MES to pH 7.5, 5 mM DTT), protease inhibitor cocktail (Sigma-Aldrich), phosphatase inhibitor cocktail 2 (Sigma-Aldrich) and phosphatase inhibitor cocktail 3 (Sigma-Aldrich) were added from stock solutions (50 μL per 10 mL of the homogenization buffer just before use). Tissue was homogenized with a Teflon Potter-Elvehjem on ice. The homogenate was filtered through a 21 μm nylon mesh, and centrifuged at 7,500 × *g* for 15 min at 4°C. Finally, the supernatant was centrifuged at 48,000 × *g* for 80 min at 4°C. The resulting pellet was the MF and stored at -80°C.

### Preparation of Tonoplast Protein

*Arabidopsis* plants were cultivated for 28 days under short day conditions (10 h light, 14 h dark) at 22°C. To achieve salt stress the plants were afterward watered using tap water containing 400 mM NaCl at day 29 and at day 31. Vacuoles/tonoplasts were isolated from these plants at day 32. To generate drought stress, watering of the corresponding plants was stopped at day 28 and vacuoles/tonoplast isolation was started when dehydration symptoms were monitored (wilting of plant leaves, about 7–8 days after termination of watering). Vacuoles and tonoplast membranes were isolated following exactly the protocol described (Schulze et al., 2012). Isolated protein (10 μg) was spiked with labeled QconCAT in a protein:QconCAT ratio of 5:1 and was jointly digested by trypsin. After desalting over C18, 5 μg of the resulting peptides were analyzed.

## Author Contributions

HP-O: performed lab experiments, analyzed the data, wrote the paper. KD: performed the salt stress experiments. OT: performed drought stress experiments, sample preparation, contributed to paper writing. HN: contributed significantly to paper writing. WS: conceived the idea and wrote main parts of the paper.

## Supplementary Material

The Supplementary Material for this article can be found online at: http://journal.frontiersin.org/article/10.3389/fpls.2016.00411

TABLE S1 **TABLE S1 | The peptides on QconCAT1 which was used as a spiked-in standard protein in this study**.

## Conflict of Interest Statement

The authors declare that the research was conducted in the absence of any commercial or financial relationships that could be construed as a potential conflict of interest.
